# Supplementary material for: Integrative multi-region molecular profiling of primary prostate cancer in men with synchronous lymph node metastasis
Source: Nat Commun. 2024 May 21;15:4341. doi: 10.1038/s41467-024-48629-y (PMC11109137; doi:10.1038/s41467-024-48629-y)
Supplement: Supplementary file 1 — Supplementary Information [file 41467_2024_48629_MOESM1_ESM.pdf]

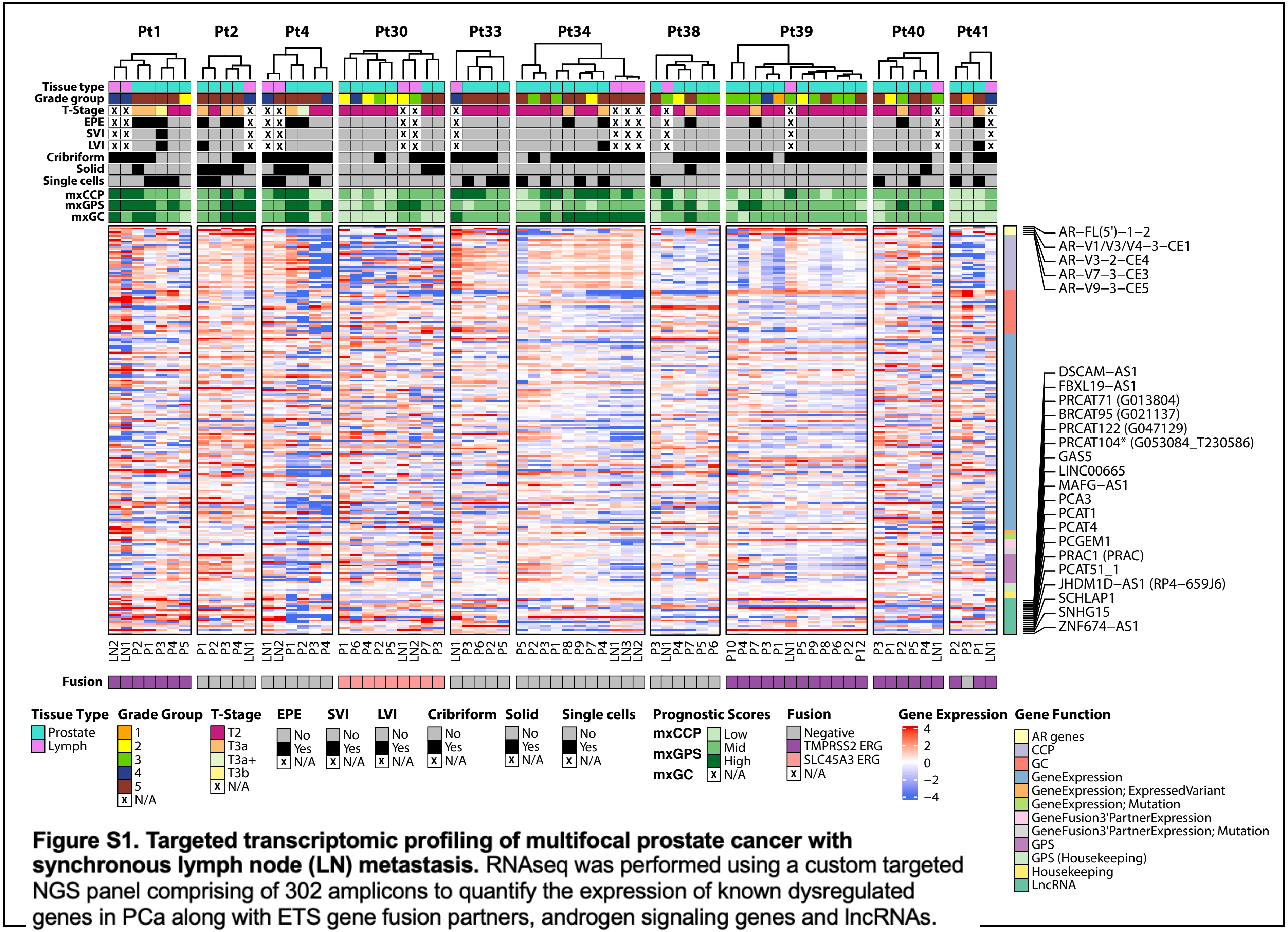

Figure S1. Targeted transcriptomic profiling of multifocal prostate cancer with synchronous lymph node (LN) metastasis. RNAseq was performed using a custom targeted NGS panel comprising of 302 amplicons to quantify the expression of known dysregulated genes in PCa along with ETS gene fusion partners, androgen signaling genes and lncRNAs. Unsupervised hierarchical clustering of 223 genes (excluding fusion amplicons) that passed QC was performed to derive expression profiles for each patient. Samples were clustered using a complete linkage method whereas genes were ordered by their function. Key *AR* genes and lncRNAs were highlighted on the heatmap.

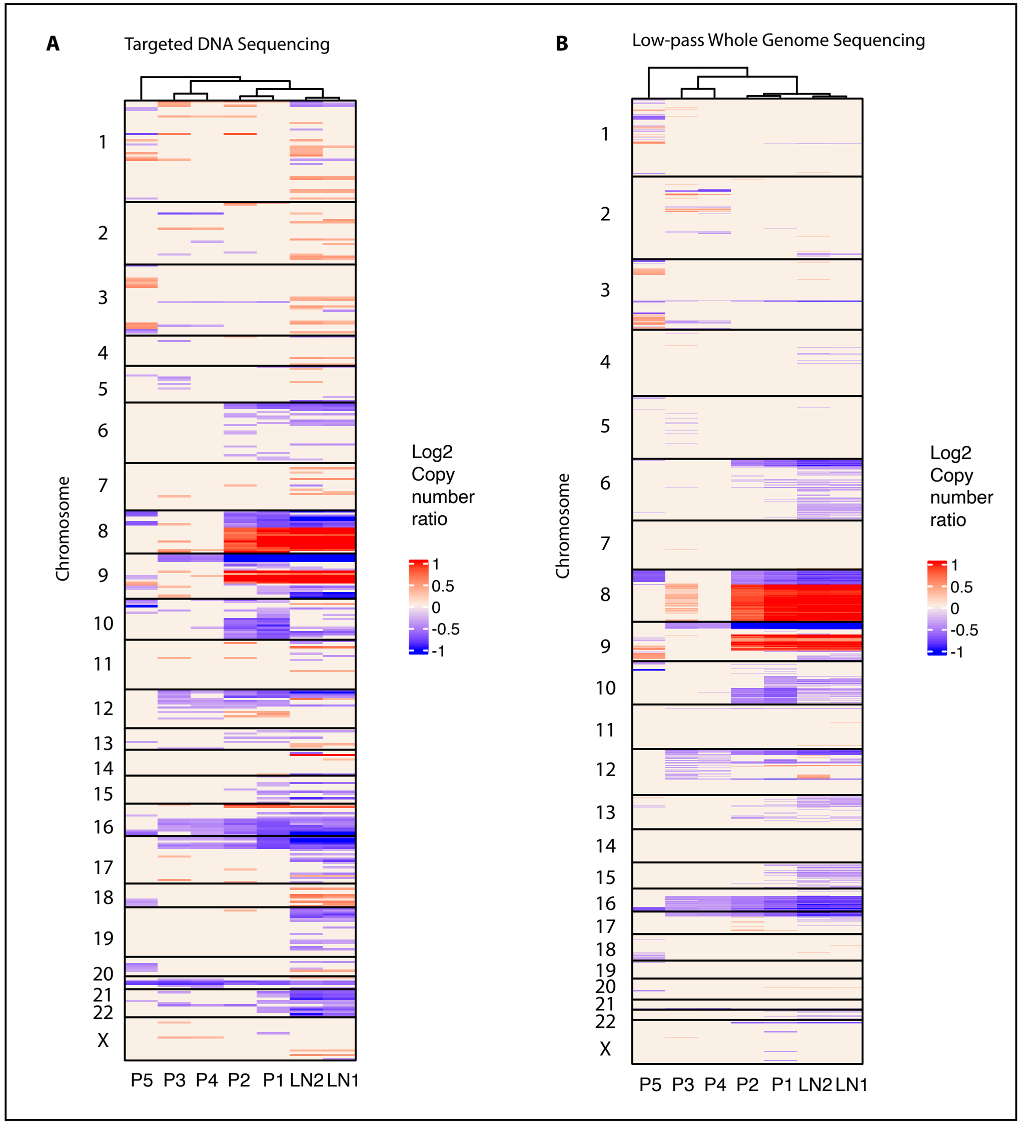

Figure S2. Comparison of copy number alterations (CNA) detected by targeted DNA NGS versus low-pass whole genome sequencing (LPWGS). To demonstrate the accuracy of our targeted DNA NGS panels for calling CNA (described in **Fig. 1**), we performed LPWGS in the samples obtained from patient #1 (n=8: 5 primary tumors, P1-P5; LN metastasis foci, LN1,2; and one normal (N) area). **A.** Unsupervised hierarchical for CNA by targeted DNA NGS as described in **Fig.** 1 is shown. **B.** CNA analysis from LPWGS data was performed using the ichorCNA package in *R* to determine log2CN values by comparing tumors to the paired normal. Bin sizes (only absolute log2CNvalue > 0.3) with losses and gains are displayed in the heatmap. Genes were ordered by the chromosome number along with their start and end positions within each chromosome. Unsupervised hierarchical clustering of the tumor and LN metastases regions was performed using complete method. Unsurprisingly, similar CNAs e.g. 8p loss and 8q gain were detected by both targeted DNA NGS (**panel A**) and LPWGS (**panel B**) approaches. Critically, primary tumor samples P1, P2 clustered together with LN metastases regions LN1, LN2 in the unsupervised hierarchical clustering of the samples in both approaches supporting the validity of the targeted DNA NGS approach utilized in the entire study cohort.

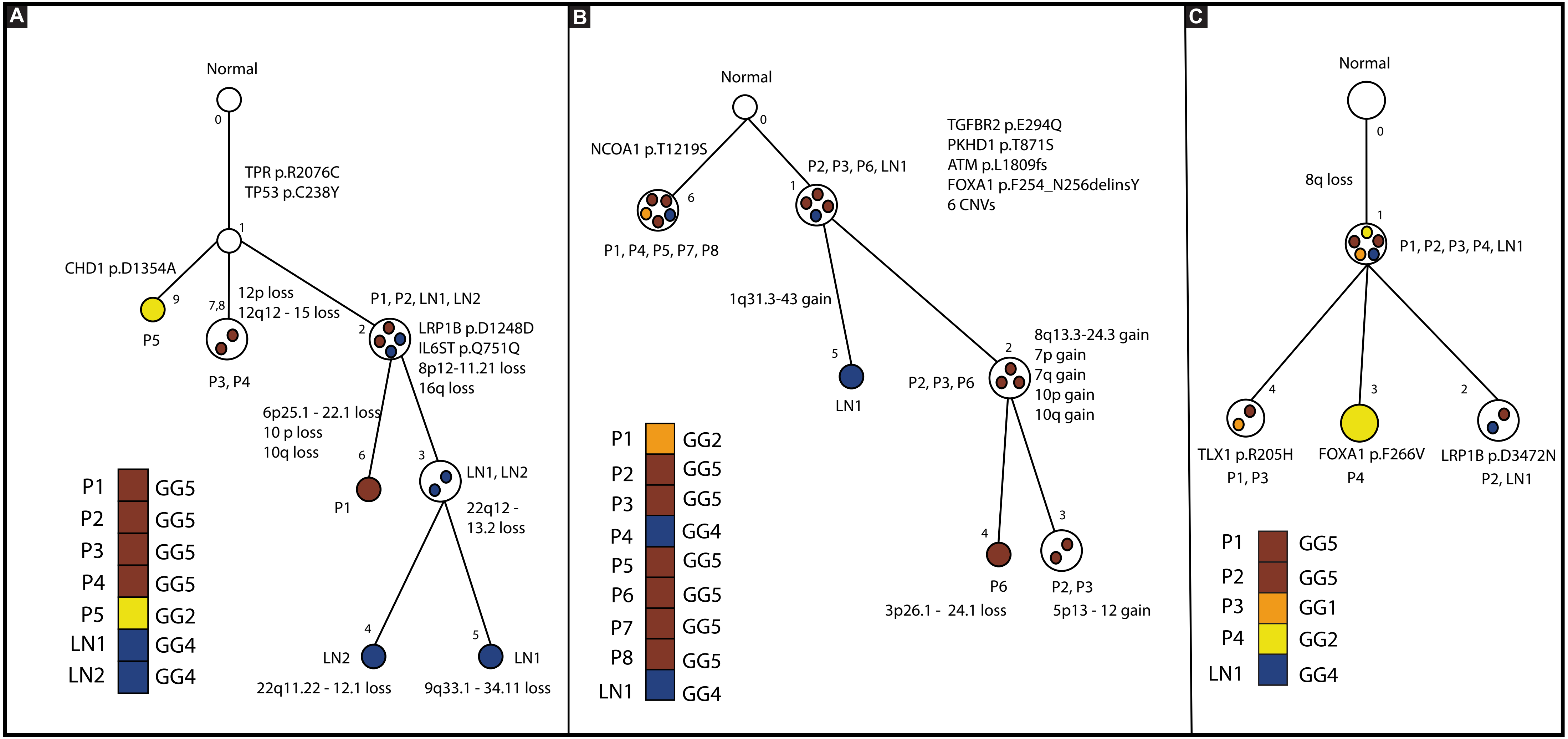

Figure S3. Phylogenetic evolutionary analysis to determine the biologically dominant nodule using the PhyloWGS method. All phylogenetic trees represented here were created from PhyloWGS and the selected tree was based on the program's metric of the most similar tree to all other trees. All trees are subclonal reconstructions with 0 indicating the normal. The corresponding cellular prevalence and cancer cell fraction (CCF) making up each node by sample are shown in **Table S2** (nodes with relatively low CCF are not shown in the figure). **A. Patient #1**: The clonal somatic variants (*LRP1B, IL6ST*) and CNAs (16q loss) splinter P1, P2, LN1 and LN2 from the other tumor areas. The two LN metastatic tumors branch off from the primary tumors based on aneuploidy (10p, 10q) or large CNAs (22q12 and 9q33). **B. Patient #33:** Multiple primary trees were found given the large amounts of genomic instability and somatic variants found in the various tumor regions. Tumors (P2, P3, P6) that gave rise to the metastatic tumor samples were mainly driven by somatic mutations in *TGFBR2, PKHD1, ATM and FOXA1*. The LN metastatic tumor separated from the other tumors mainly by a gain in 1q31-43 and *LRP1B* mutation. **C. Patient #41**: This patient had low amounts of somatic mutation and CNAs. P2, P4 and LN1 all shared an 8q loss and then were differentiated by mutations in *FOXA1* (P4) and *LRP1B* (P2 and LN1). P1 was filtered out by the algorithm despite having the *TLX1* mutation because a clonal loss of 8q was not found in this sample.

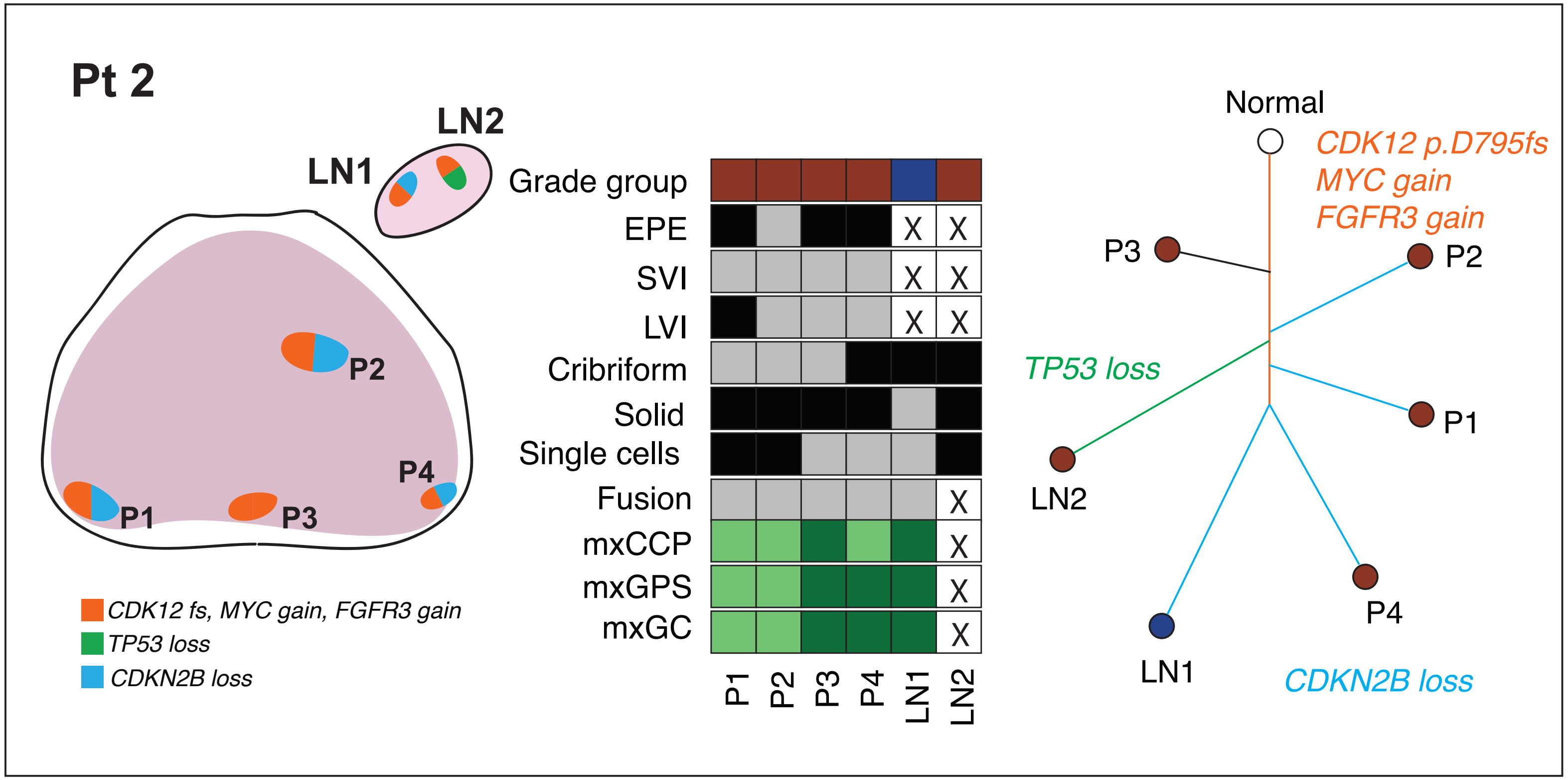

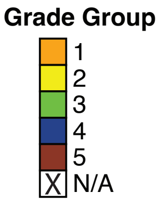

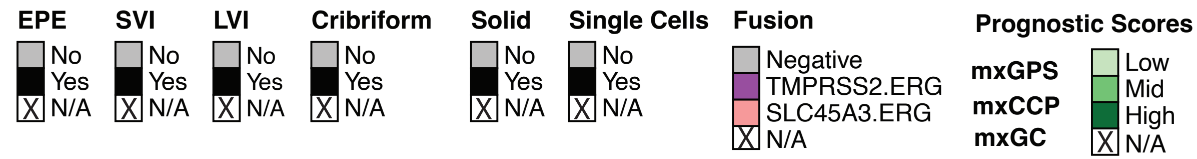

Figure S4-10. Patient level phylogenetic evolutionary analysis to determine the biologically dominant nodule. Each patient figure included in this supplement is described in the following order: **Left panel.** The spatial location of each tumor sample on the radical prostatectomy specimen is annotated on a diagram of the prostate; **Middle panel.** Relevant clinicopathologic variables and gene signatures are annotated for each tumor region. **Right panel.** Evolutionary analysis was performed using neighbor-joining method by feeding DNA mutations, significant CNA, and gene fusion status (present/absent) into the algorithm. Phylogenetic trees were constructed with R *phangorn* package using neighbor joining method.

Figure S4. In **patient #2**, 4 primary (all GG 5) and 2 LN metastatic prostate cancer regions were analyzed. All tumor regions with available data were ETS gene fusion negative. Bi-allelic inactivating *CDK12* mutations, *MYC* and *FGFR3* gains were detected in all tumor regions. *CDKN2B* loss was observed in primary tumor regions P1, P2, and P4 as well as LN1. The phylogenetic tree suggests that P4 (with EPE, cribriform pattern, and solid cell pattern) closely resembles LN1 focus and a possible separate trajectory for LN2 which subsequently developed *TP53* loss.

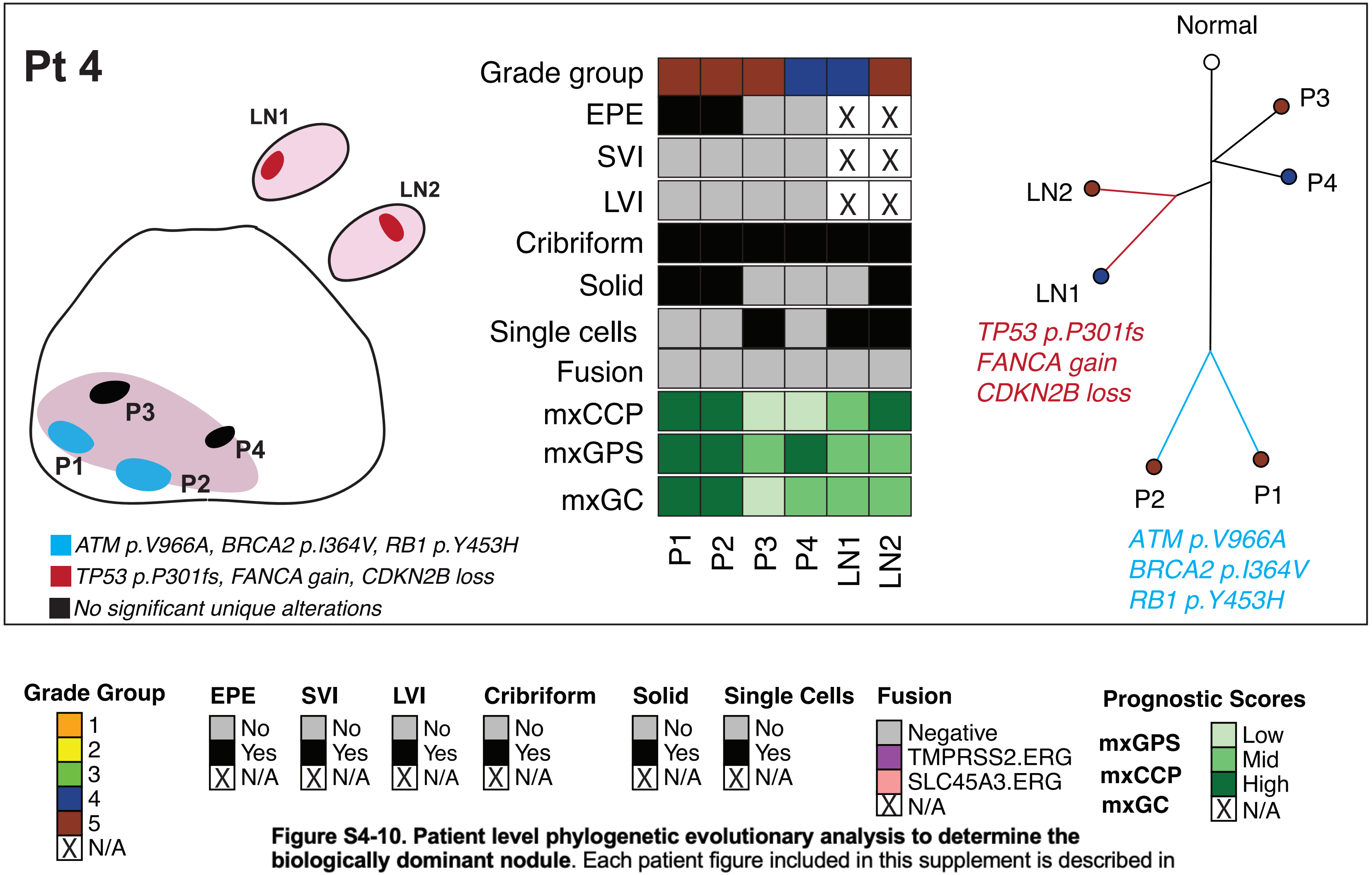

Figure S4-10. Patient level phylogenetic evolutionary analysis to determine the biologically dominant nodule. Each patient figure included in this supplement is described in the following order: **Left panel.** The spatial location of each tumor sample on the radical prostatectomy specimen is annotated on a diagram of the prostate; **Middle panel.** Relevant clinicopathologic variables and gene signatures are annotated for each tumor region. **Right panel.** Evolutionary analysis was performed using neighbor-joining method by feeding DNA mutations, significant CNA, and gene fusion status (present/absent) into the algorithm. Phylogenetic trees were constructed with R *phangorn* package using neighbor joining method.

Figure S5. In **patient #4**, a total of 4 primary (GG 4-5) and 2 LN metastases regions were analyzed. All tumor regions inclusive of LN metastases were ETS gene fusion negative. A *TP53* frameshift mutation, *FANCA* gain, and *CDKN2B* loss were detected only in LN metastasis regions. Primary tumor regions P1 and P2 harbored several diverse driver mutations which were not detected in other regions. Phylogenetic tree analysis suggests that P3 and P4 likely gave rise to LN metastatic regions LN1 and LN2.

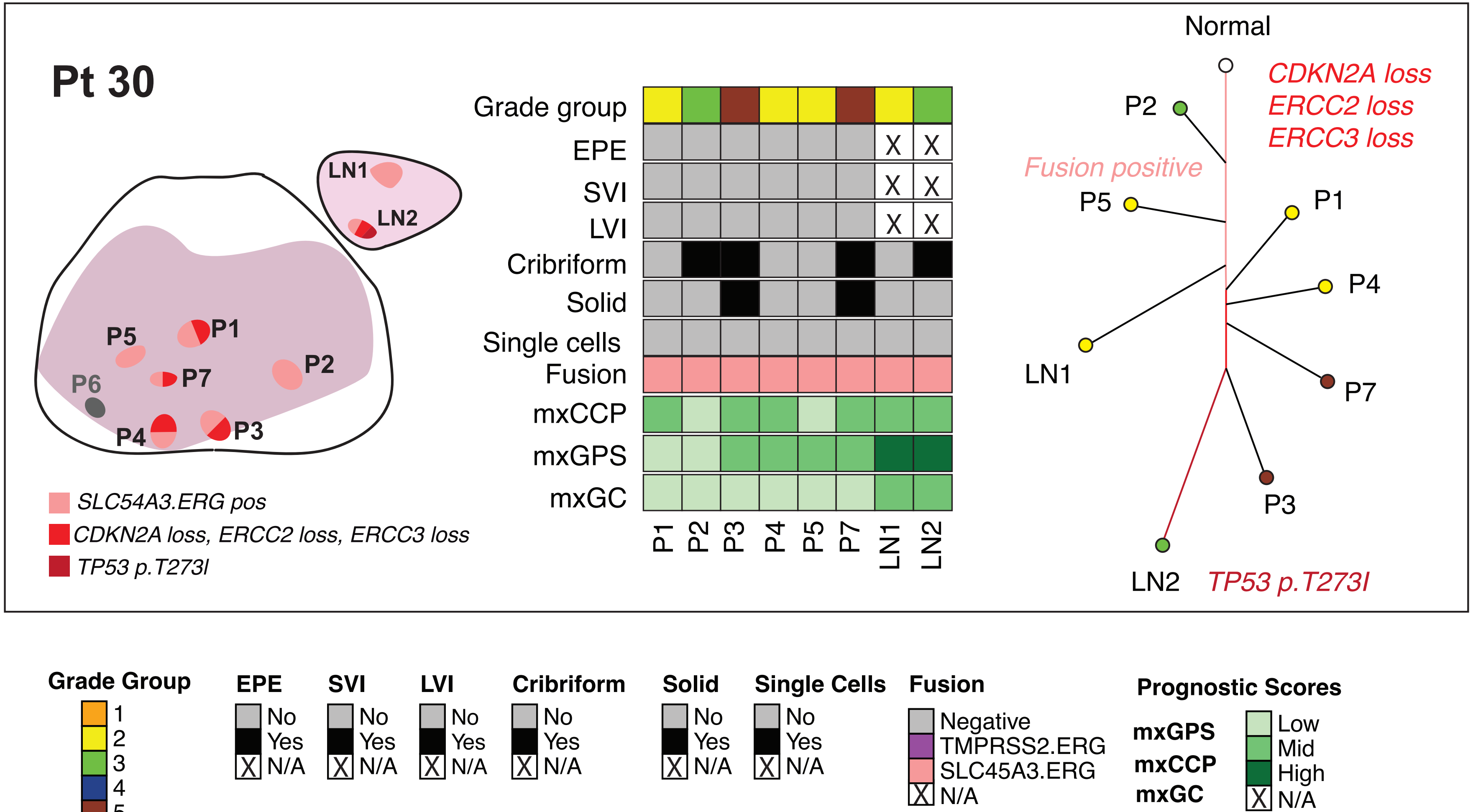

Figure S4-10. Patient level phylogenetic evolutionary analysis to determine the biologically dominant nodule. Each patient figure included in this supplement is described in the following order: **Left panel.** The spatial location of each tumor sample on the radical prostatectomy specimen is annotated on a diagram of the prostate; **Middle panel.** Relevant clinicopathologic variables and gene signatures are annotated for each tumor region. **Right panel.** Evolutionary analysis was performed using neighbor-joining method by feeding DNA mutations, significant CNA, and gene fusion status (present/absent) into the algorithm. Phylogenetic trees were constructed with R *phangorn* package using neighbor joining method.

Figure S6. In **patient #30,** a total of 7 primary tumor (GG 2 – 5) and 2 LN metastases regions were analyzed. All regions inclusive of the LN metastases were positive for *SLC45A3:ERG* gene fusion. *CDKN2A*, *ERCC2* and *ERCC3* losses were observed in primary tumor regions P1, P3, P4, P7 and LN metastasis region LN2. Additionally, *TP53* mutation was detected only in LN metastasis region LN2. In the phylogenetic analyses, however, only primary tumor regions P3 closely resembles LN2. (*Genomic data was not available for primary tumor region P6).

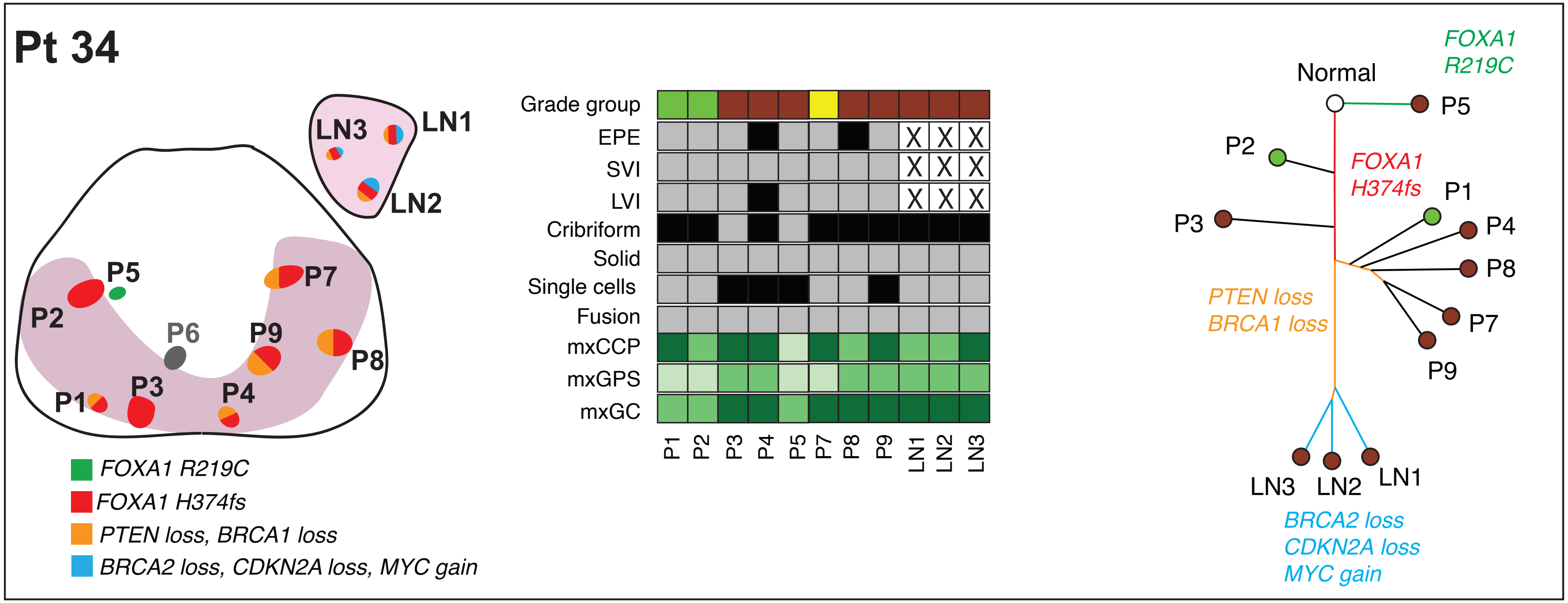

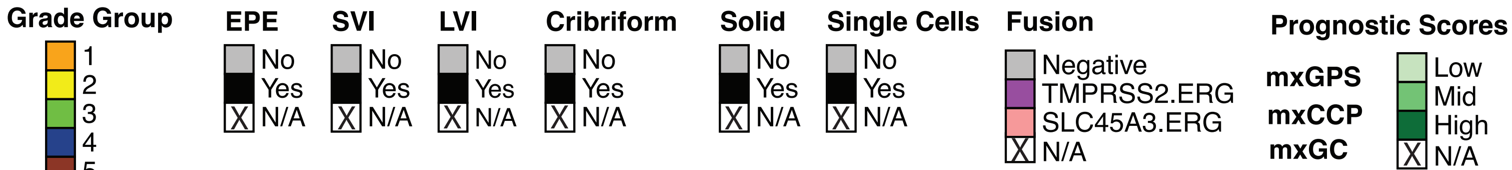

Figure S4-10. Patient level phylogenetic evolutionary analysis to determine the biologically dominant nodule. Each patient figure included in this supplement is described in the following order: **Left panel.** The spatial location of each tumor sample on the radical prostatectomy specimen is annotated on a diagram of the prostate; **Middle panel.** Relevant clinicopathologic variables and gene signatures are annotated for each tumor region. **Right panel.** Evolutionary analysis was performed using neighbor-joining method by feeding DNA mutations, significant CNA, and gene fusion status (present/absent) into the algorithm. Phylogenetic trees were constructed with R *phangorn* package using neighbor joining method.

Figure S7. In **patient #34,** a total of 9 primary tumor (GG 2-5) and 3 LN metastasis tumor regions were analyzed. All tumor regions were *ETS* gene fusion negative. P6 region was excluded from phylogenetic analysis due to low tumor content. A *FOXA1* frameshift mutation was observed in all regions except for P5. A different *FOXA1 R219C* mutation was observed in P5. This patient's samples exhibit genomic complexity with CNA (*BRCA1* and *PTEN* losses) present in P1, P4, P7, P8, P9, LN1, LN2 and LN3 but absent in P2, P3 and P5 suggesting two branches of clonal evolution. Notably, LN1, LN2 and LN3 displayed genomic homogeneity with additional *BRCA2* and *CDKN2A* losses as well as *MYC* gain. Regions P1, P4, P7, P8, P9 thus represent the dominant cancer area resulting in LN metastasis.

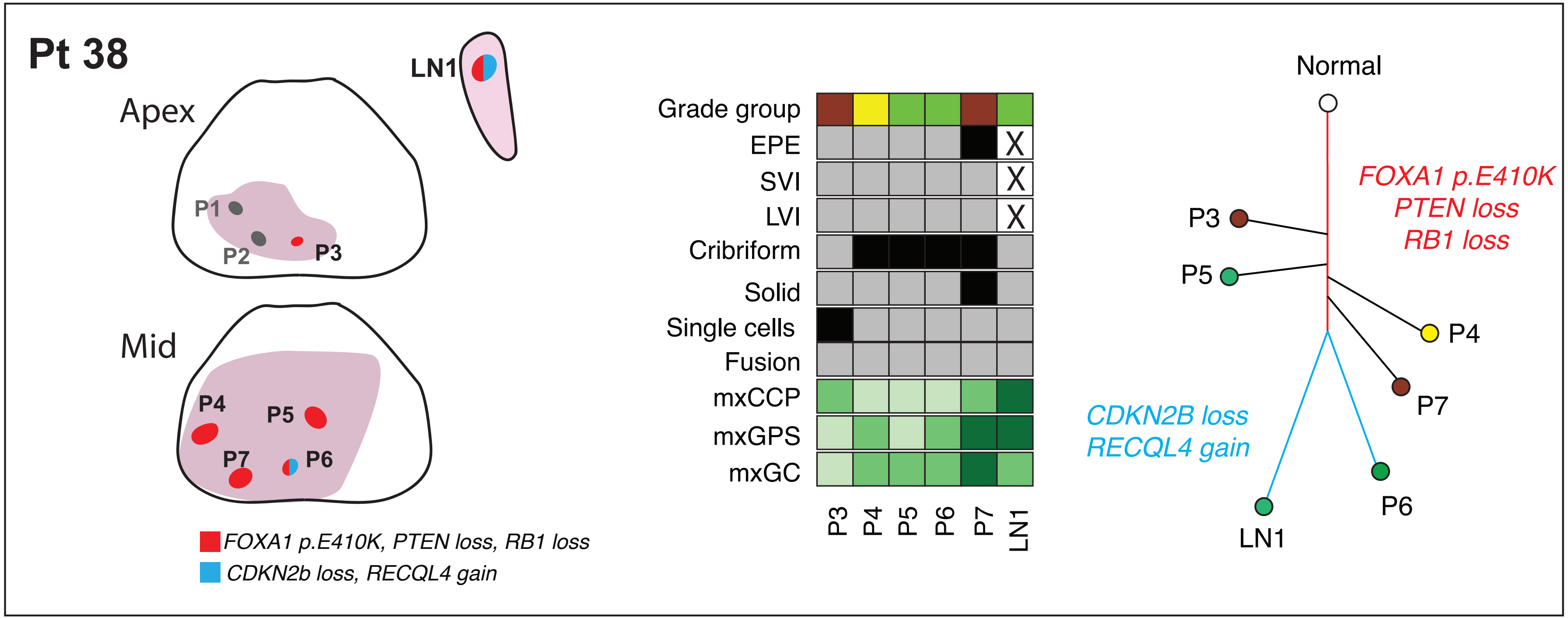

Figure S4-10. Patient level phylogenetic evolutionary analysis to determine the biologically dominant nodule. Each patient figure included in this supplement is described in the following order: **Left panel.** The spatial location of each tumor sample on the radical prostatectomy specimen is annotated on a diagram of the prostate; **Middle panel.** Relevant clinicopathologic variables and gene signatures are annotated for each tumor region. **Right panel.** Evolutionary analysis was performed using neighbor-joining method by feeding DNA mutations, significant CNA, and gene fusion status (present/absent) into the algorithm. Phylogenetic trees were constructed with R *phangorn* package using neighbor joining method.

Figure S8. In **patient #38**, a total of 7 primary tumor (GG 2-5) and 1 LN metastatic cancer regions were analyzed. P1, P2 and P3 were in the apex whereas P4, P5, P6 and P7 were in mid prostate. All regions were ETS fusion negative. A *FOXA1* mutation along with *PTEN* and *RB1* losses were detected in all tumor regions except for P1 and P2 which had low tumor content and therefore, were removed from phylogenetic analysis. Among P3, P4, P5, P6, P7 and LN1, only P6 and LN1 displayed similar alterations such as *CDKN2B* loss and *RECQL4* gain, suggesting a common clonal origin.

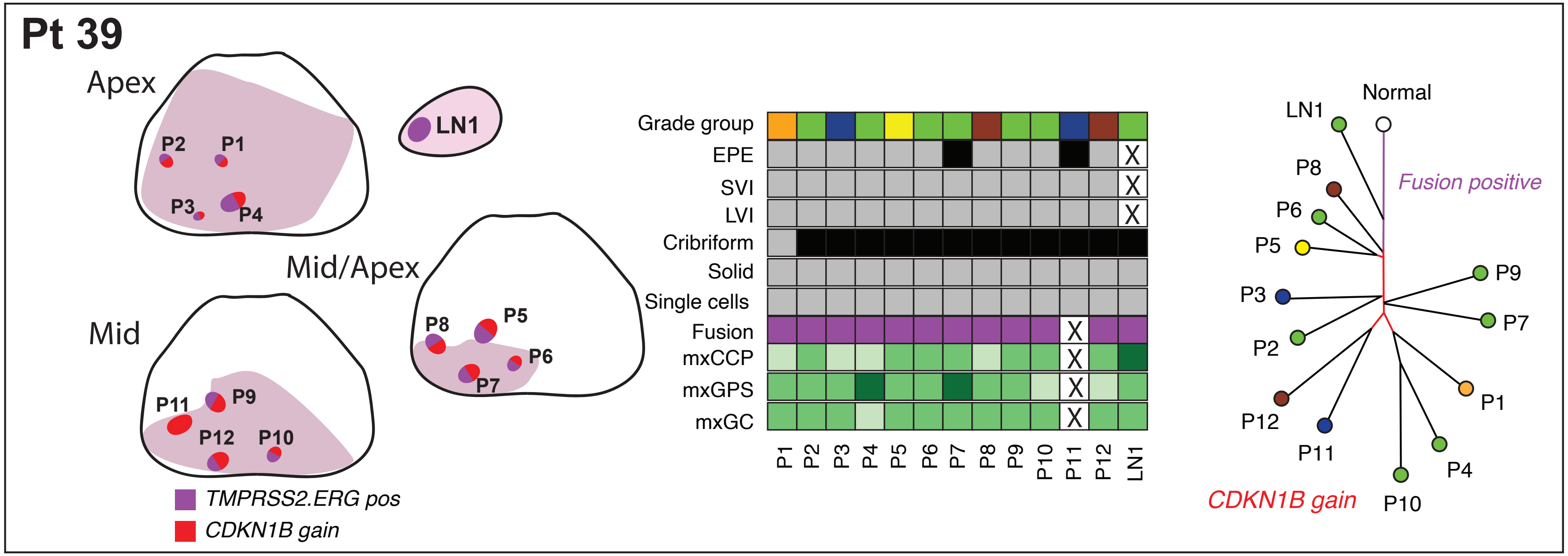

Figure S4-10. Patient level phylogenetic evolutionary analysis to determine the biologically dominant nodule. Each patient figure included in this supplement is described in the following order: **Left panel.** The spatial location of each tumor sample on the radical prostatectomy specimen is annotated on a diagram of the prostate; **Middle panel.** Relevant clinicopathologic variables and gene signatures are annotated for each tumor region. **Right panel.** Evolutionary analysis was performed using neighbor-joining method by feeding DNA mutations, significant CNA, and gene fusion status (present/absent) into the algorithm. Phylogenetic trees were constructed with R *phangorn* package using neighbor joining method.

Figure S9. In **patient #39**, a total of 12 primary tumor (GG 1-5) and 1 LN regions were analyzed. Regions P1, P2, P3 and P4 were in the apex; P5, P6, P7 and P8 extended from the apex to mid prostate, whereas regions P9, P10, P11 and P12 were in the mid prostate. All tumor regions were *TMPRSS2:ERG* gene fusion positive except for P11 (RNAseq data did not meet QC metrics). No significant somatic mutations were detected in this patient. *CDKN1B* gain was detected in all primary tumor regions except for LN1. The dominant tumor region giving rise to LN metastasis is unknown in this patient.

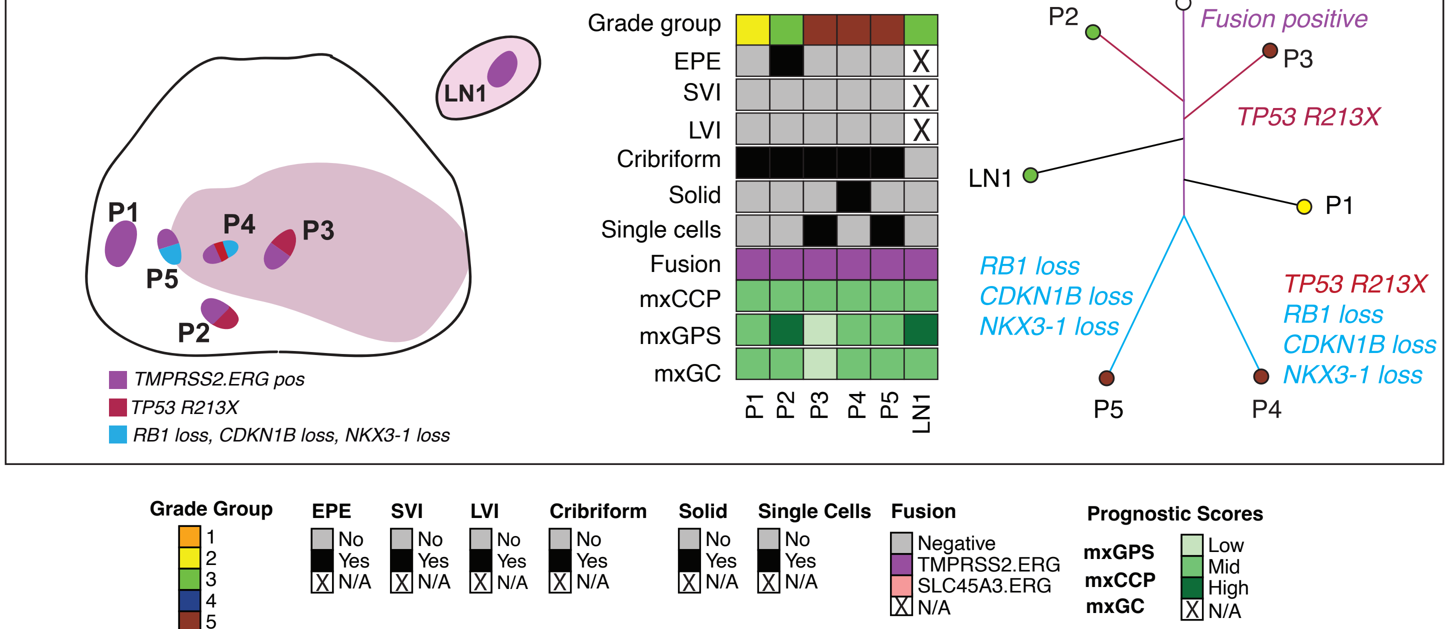

Figure S4-10. Patient level phylogenetic evolutionary analysis to determine the biologically dominant nodule. Each patient figure included in this supplement is described in the following order: **Left panel.** The spatial location of each tumor sample on the radical prostatectomy specimen is annotated on a diagram of the prostate; **Middle panel.** Relevant clinicopathologic variables and gene signatures are annotated for each tumor region. **Right panel.** Evolutionary analysis was performed using neighbor-joining method by feeding DNA mutations, significant CNA, and gene fusion status (present/absent) into the algorithm. Phylogenetic trees were constructed with R *phangorn* package using neighbor joining method.

Figure S10. In **patient #40**, a total of 5 primary tumor (GG 2-5) and 1 LN metastasis regions

Figure S4-10. Patient level phylogenetic evolutionary analysis to determine the biologically dominant nodule. Each patient figure included in this supplement is described in the following order: **Left panel.** The spatial location of each tumor sample on the radical prostatectomy specimen is annotated on a diagram of the prostate; **Middle panel.** Relevant clinicopathologic variables and gene signatures are annotated for each tumor region. **Right panel.** Evolutionary analysis was performed using neighbor-joining method by feeding DNA mutations, significant CNA, and gene fusion status (present/absent) into the algorithm. Phylogenetic trees were constructed with R *phangorn* package using neighbor joining method.

Figure S10. In **patient #40,** a total of 5 primary tumor (GG 2-5) and 1 LN metastasis regions were analyzed. All tumor regions were *TMPRSS2:ERG* gene fusion positive. A *TP53* somatic mutation was detected in regions P2, P3 and P4. *CDKN1B, NKX3-1 and RB1* losses were detected in regions P4 and P5. The dominant tumor region giving rise to LN metastasis is unknown in this patient.
