# Supplementary material for: Integrative multi-region molecular profiling of primary prostate cancer in men with synchronous lymph node metastasis
Source: Nat Commun. 2024 May 21;15:4341. doi: 10.1038/s41467-024-48629-y (PMC11109137; doi:10.1038/s41467-024-48629-y)
Supplement: Supplementary file 3 — Reporting Summary [file 41467_2024_48629_MOESM3_ESM.pdf]

nature portfolio

Corresponding author(s): Udit Singhal, Simpa S Salami

Last updated by author(s): 3/8/2024

Reporting Summary

Nature Portfolio wishes to improve the reproducibility of the work that we publish. This form provides structure for consistency and transparency in reporting. For further information on Nature Portfolio policies, see our Editorial Policies and the Editorial Policy Checklist.

Statistics

For all statistical analyses, confirm that the following items are present in the figure legend, table legend, main text, or Methods section.

n/a	Confirmed	
☐	☒	The exact sample size (*n*) for each experimental group/condition, given as a discrete number and unit of measurement
☐	☒	A statement on whether measurements were taken from distinct samples or whether the same sample was measured repeatedly
☒	☐	The statistical test(s) used AND whether they are one- or two-sided<br>*Only common tests should be described solely by name; describe more complex techniques in the Methods section.*
☐	☒	A description of all covariates tested
☐	☒	A description of any assumptions or corrections, such as tests of normality and adjustment for multiple comparisons
☐	☒	A full description of the statistical parameters including central tendency (e.g. means) or other basic estimates (e.g. regression coefficient) AND variation (e.g. standard deviation) or associated estimates of uncertainty (e.g. confidence intervals)
☒	☐	For null hypothesis testing, the test statistic (e.g. *F*, *t*, *r*) with confidence intervals, effect sizes, degrees of freedom and *P* value noted<br>*Give P values as exact values whenever suitable.*
☒	☐	For Bayesian analysis, information on the choice of priors and Markov chain Monte Carlo settings
☐	☒	For hierarchical and complex designs, identification of the appropriate level for tests and full reporting of outcomes
☒	☐	Estimates of effect sizes (e.g. Cohen's *d*, Pearson's *r*), indicating how they were calculated

Our web collection on statistics for biologists contains articles on many of the points above.

Software and code

Policy information about availability of computer code

Data collection	Data collection and curation was performed with R statistical software v4.1.0
Data analysis	All downstream data analyses were performed using R statistical software.

For manuscripts utilizing custom algorithms or software that are central to the research but not yet described in published literature, software must be made available to editors and reviewers. We strongly encourage code deposition in a community repository (e.g. GitHub). See the Nature Portfolio guidelines for submitting code & software for further information.

Data

Policy information about availability of data

All manuscripts must include a data availability statement. This statement should provide the following information, where applicable:

- Accession codes, unique identifiers, or web links for publicly available datasets
- A description of any restrictions on data availability
- For clinical datasets or third party data, please ensure that the statement adheres to our policy

The bam files generated in this study have been deposited to the European Nucleotide Archive (ENA) database under accession code PRJEB72307. The data is publicly available and access can be obtained at ENA website [https://www.ebi.ac.uk/ena/browser/view/PRJEB72037]. The R code used for the analysis has been deposited to github [https://github.com/srinew/PCA-LNMETS].

Research involving human participants, their data, or biological material

Policy information about studies with human participants or human data. See also policy information about sex, gender (identity/presentation), and sexual orientation and race, ethnicity and racism.

Reporting on sex and gender	Prostate cancer is a disease of male sex, and all patients in this study self-reported as male.
Reporting on race, ethnicity, or other socially relevant groupings	No reported or analysis by race/ethnicity was performed.
Population characteristics	described in text:<br><br>With local Institutional Review Board approval (IRB #: HUM00042749) we assembled a retrospective, multi-institutional, non-consecutive cohort of patients with primary prostate cancer with LN metastases. First, from a prior transcriptomic analysis of samples chosen to represent the clinical continuum of prostate cancer by our group7, we identified six patients with synchronous LN metastases at the time of radical prostatectomy (cohort 1). Second, we assembled an additional non-consecutive cohort of twelve patients with large or multifocal prostate cancer with synchronous LN metastases from participating institutions (cohort 2). From a combined cohort of 18 patients (103 primary tumor and 28 LN metastasis samples), 10 patients (65 primary tumor and 16 LN metastasis samples) met experimental and analytic QC metrics as well as had sufficient quality data for phylogenetic analysis. Complete sample information is provided in Table S1. All profiled samples were evaluated by a board-certified Anatomic Pathologist (S.A.T., A.M.U., R.L.) with combined experience in prostate and molecular pathology who assigned a Gleason score, GG and outlined tumor regions within the specimens for molecular profiling.
Recruitment	Written informed consent was not needed or obtained for this retrospective analysis of archival tissue specimens. Participants were not compensated for participation in the study, as archival tissue specimens were analyzed in a retrospective fashion.
Ethics oversight	With local Institutional Review Board approval (IRB #: HUM00042749) we assembled a retrospective, multi-institutional, non-consecutive cohort of men with primary prostate cancer with LN metastases. All study aspects were performed in accordance with the Good Clinical Practice Guidelines and Declaration of Helsinki.

Note that full information on the approval of the study protocol must also be provided in the manuscript.

Field-specific reporting

Please select the one below that is the best fit for your research. If you are not sure, read the appropriate sections before making your selection.

☒ Life sciences ☐ Behavioural & social sciences ☐ Ecological, evolutionary & environmental sciences

For a reference copy of the document with all sections, see nature.com/documents/nr-reporting-summary-flat.pdf

Life sciences study design

All studies must disclose on these points even when the disclosure is negative.

Sample size	No sample size or power calculation was performed, as no findings were used to predict or prognosticate long-term outcomes. No minimum sample size was needed for evaluation/discovery of genes involved in multi-focal prostate cancer to identify patterns of synchronous lymph node metastasis.
Data exclusions	Those who did not meetexperimental and analytic QC metrics, or did not have sufficient quality data for phylogenetic analysis were excluded.
Replication	Analyses were conducted in triplicate as necessary. All attempts at replication were successful unless otherwise specifically outlined in the study.
Randomization	This was not a clinical trial or prospective study, therefore no randomization was needed or performed.
Blinding	This was not a clinical trial or prospective study, therefore no blinding was needed or performed.

Reporting for specific materials, systems and methods

We require information from authors about some types of materials, experimental systems and methods used in many studies. Here, indicate whether each material, system or method listed is relevant to your study. If you are not sure if a list item applies to your research, read the appropriate section before selecting a response.

Materials & experimental systems

n/a	Involved in the study
☒	☐ Antibodies
☒	☐ Eukaryotic cell lines
☒	☐ Palaeontology and archaeology
☒	☐ Animals and other organisms
☐	☒ Clinical data
☒	☐ Dual use research of concern
☒	☐ Plants

Methods

n/a	Involved in the study
☒	☐ ChIP-seq
☒	☐ Flow cytometry
☒	☐ MRI-based neuroimaging

Clinical data

Policy information about clinical studies

All manuscripts should comply with the ICMJE guidelines for publication of clinical research and a completed CONSORT checklist must be included with all submissions.

Clinical trial registration	N/A
Study protocol	(IRB #: HUM00042749
Data collection	retrospective, multi-institutional, non-consecutive cohort of patients with primary prostate cancer with LN metastases, as well as an additional non-consecutive cohort of twelve patients with large or multifocal prostate cancer with synchronous LN metastases from participating institutions
Outcomes	N/A

Plants

Seed stocks	N/A
Novel plant genotypes	N/A
Authentication	N/A
